# Demanding and rewarding: Midwives experiences of starting a continuity of care project in rural Sweden

**DOI:** 10.18332/ejm/133573

**Published:** 2021-03-22

**Authors:** Birgitta Larsson, Li Thies-Lagergren, Annika Karlström, Ingegerd Hildingsson

**Affiliations:** 1 Department of Health Promoting Science, Sophiahemmet University, Stockholm, Sweden; 2 Department of Health Sciences, Lund University, Lund, Sweden; 3 Department of Obstetrics and Gynaecology, Helsingborg Lasarett, Helsingborg, Sweden; 4 Department of Nursing, Mid Sweden University, Sundsvall, Sweden; 5 Department of Women’s and Children’s Health, Uppsala University, Uppsala, Sweden

**Keywords:** caseload, continuity, interview, midwifery, rural area

## Abstract

**INTRODUCTION:**

The closure of a local labor ward enhanced the possibility to initiate a continuity of midwifery care model project. Continuity models of midwifery care are a cornerstone in midwifery and women-centered care, mainly accessible in metropolitan areas. Australian studies have found continuity of midwifery care to work well in rural areas. The aim of this study is to describe midwives’ experiences of developing and working in a continuity of midwifery model of care in a rural setting in Sweden.

**METHODS:**

We used a qualitative longitudinal interview with a participatory action research approach. The project was subjected to changes over time to allow the midwives to provide the best care options and to develop a model suitable for a rural area in northern Sweden.

**RESULTS:**

The overarching theme, ‘Developing a continuity model of midwifery care – demanding and rewarding with new insights’, was based on three themes: 1) A challenging but evolving start, 2) Varying views within the midwifery group, and 3) Visions for the future. It was revealed that the midwives had to handle the grief process of the closure of the labor ward alongside their enthusiasm of being part of a continuity of midwifery care model project.

**CONCLUSIONS:**

The establishment of the model in light of the labor ward closure was associated with conflict within the community and this had implications for the midwives. Midwives who are attracted to work in continuity models need to understand and incorporate the prerequisites of such models. In addition, long commuting to a labor ward requires enough midwives to maintain safety and security for the women at all times.

## INTRODUCTION

Continuity models of midwifery care are an approach and cornerstone in midwifery and women-centered care^1^. The rationale behind such an approach is to provide individualized care from a known midwife, or a smaller group of midwives, throughout pregnancy and birth^2^. Continuity of care is known to create positive experiences of pregnancy and birth^2-4^. Women are more satisfied with a continuity model of midwifery care, since it offers personalized care in which the midwife–woman relationship is central^5,6^.

Continuity models of midwifery care have afforded benefits to both women and the midwifery workforce. A systematic review and meta-analysis were recently published^7^ with the aim to determine the prevalence, levels and factors related to the burnout syndrome among midwives. It was reported that burnout levels varied between different care models. Continuity models, such as the caseload midwifery model^8^, presented lower levels of burnout than traditional models. Factors such as autonomy, care continuity and work schedule flexibility were beneficial. Being able to balance work and family life in such a model led to the midwives feeling more satisfied with their work, despite offering increased access to care for the women^7^.

The extent of continuity models of midwifery care is increasing, furthermost in countries where midwives have a central role in childbirth^9-12^. While these models are mainly accessible in metropolitan areas, there is evidence that continuity of midwifery care works well in rural areas^13^. One Australian integrative literature review^14^ and one study based on a retrospective clinical audit and narratives from midwives working in a rural Australian caseload model^15^ concluded that there is evidence to support caseload midwifery and its implementation in a rural setting^14,15^.

Haines et al.^15^ showed that clinical outcomes in a rural area were safe, sustainable, and comparable to national data over almost two decades. In another study, the development of a rural maternity service was described with a collaborative team approach, midwifery leadership and a focus on continuity of care^16^.

In Sweden, where midwives are the primary caregivers for women with uncomplicated pregnancies and births, continuity during the antenatal period is good, with the majority of women meeting 1–2 midwives during pregnancy^17^. Continuity throughout antenatal, intrapartum and postpartum periods is, however, rare and mainly offered in urban areas. This study reports a continuity of midwifery care project in a rural area of Sweden after the closing of the labor ward at a small hospital. The aim was to describe midwives’ experiences of developing and working in a continuity of midwifery model of care in a rural setting in Sweden.

## METHODS

### Design

A qualitative longitudinal interview study with a participatory action research approach was employed, in that the principles of democracy, participation, reflection and change were applied^18^. The project was subjected to changes over time to provide the midwives with opportunities to provide the best care options and to develop a model suitable for a rural area.

### Study context

The study was carried out in the middle-north part of Sweden, in a rural inland town in a larger region. The region is one of 21 regions in Sweden, governed by elected politicians who are responsible for providing healthcare for 245000 inhabitants. It is a geographical area with occasionally severe winter weather for about five months a year.

The town has a county district hospital that used to have a labor ward with 300–350 annual births. In February 2017, due to cutbacks, regional politicians closed the labor ward, leaving pregnant women in labor to travel 100 to 120 km to reach one of the remaining two hospitals in the region. Women could choose to give birth in a county hospital in the southern part of the region with a labor ward with an annual birth rate of 1600–1700 that also provides specialist care. Another option is a county district hospital managing 600–700 annual births at the labor ward situated in a middle-sized town in the northern part of the region.

An administrative re-organization of the hospitals in the region together with the closure of the labor ward provoked a protest among the inhabitants in the rural town. The day after the labor ward closed, people from the town occupied the hospital entrance all day, during the project period and beyond. The message from the occupiers was clear, they wanted the labor ward to re-open. This protest gained a lot of media attention but had no impact on the political decision.

During the same time as the closure of the labor ward, several governmental attempts were initiated to strengthen childbirth care in Sweden due to a national lack of midwives, as midwives were leaving the profession and claims from the consumer groups^19^. For several years, all regions in Sweden gained financial support with the aim of educating more midwives, increasing staff at the labor wards, initiating project development emphasizing continuity of care and developing other relevant initiatives^20^. The closing of the labor ward was the origin of the present continuity of care project, enabled by the governmental initiative.

### Recruitment of midwives to the project

In December 2016, shortly after the decision was made to close the labor ward, all midwives working in the labor ward, as well as the midwives working with antenatal care, were informed about the continuity project. The midwives were asked to express their interest in participating in the project, and those interested were thereafter interviewed about their expectations. Four midwives were employed. They were between 38 to 60 years old and had a working experience from 5 to 35 years, mainly in intrapartum and postpartum care.

Every fortnight, the main project leader, who was based in the county hospital in the southern part of the region, met with the group in conjunction with the local manager in the rural town and the principal investigator (IH) for the research. The working model was discussed, and plans were made for the recruitment of participants, examination rooms and offices, and practical issues, e.g. how and when to travel to the labor wards at the two hospitals, equipment and working schedules. A study visit was made to Denmark to see how midwives worked in a caseload model. The midwives had to update the routines for providing antenatal care, and they also spent time at the two labor wards in the regions to familiarize themselves with the staff and the environment.

### Organization of the continuity of midwifery model of care in the project

When the on-call service started, 1 August 2017, four midwives worked in the model (one full-time, three part-time). From 1 August 2017 to 30 April 2018, one of the midwives was on-call in her home four days in a row on weekdays every fourth week (7 a.m. to 11 p.m.) and three days every fourth weekend. One week was free, and during the remaining weeks, antenatal care was the main responsibility. After nine months (1 May 2018 to 30 June 2019), the group of midwives wanted to change the on-call system, which resulted in their being placed at the local hospital during their on-call shifts starting at 7 a.m. If there was no woman in labor, the midwife assisted colleagues or worked with administration. The midwives also changed the long on-call periods to be on call one day each on weekdays. This change affected the extent of continuity for the women^21^. An overview of the key features is shown in Table 1.

**Table 1:** Key features of the midwifery continuity model of care

1 February 2017	Four midwives employed in the project at start
February 2017–June 2019	Regular project meetings monthly
February–April 2017	Organizing rooms, equipment and work schedules
March 2017	Study visit caseload model in Denmark
March–April 2017	Introduction to antenatal care
March–May 2017	Introduction on two labor wards
1 August 2017–30 June 2019	Recruitment of pregnant women with a due date between 1 August 2017 and 30 June 2019
1 August 2017	On-call service started, midwives based at home during on-call days
1 May 2018	Change in on-call schedule, midwives based in the antenatal clinic
June 2019	End of research project

### Data collection

The midwives in the continuity model of midwifery care project were interviewed on three occasions during the project period. The first occasion took place in September 2017, seven months after the start of the project and one month after the on-call service had begun. This first occasion was conducted as group interviews with the midwives divided into two groups. Two of the researchers (AK and BL) performed both interviews together, and the average time of the interviews was one hour.

In the middle of the project, the midwives were interviewed for the second time, now through individual interviews. At this time, there had been some changes in the group, as two midwives were no longer a part of the project, and one new midwife had been recruited. There was one face-to-face interview, and three interviews were performed through telephone. The reason for the different methods was related to practical issues and the midwives’ own choices.

The third occasion of interviews took place when the project had reached the final date. At this time, four of the project midwives were individually interviewed face-to-face.

The same researchers (AK and BL) as the first group interviews carried out the individual interviews, and the time for each interview was approximately one hour. All interviews were digitally recorded after permission was received from the midwives and were consecutively transcribed verbatim by the same researchers that performed the interviews.

### Data analysis

For the analysis of the interview data, thematic analysis following the model of Braun and Clark^22^ was used with an inductive approach focusing on the semantic context. This approach was chosen since the aim of the interviews was to describe the midwives’ views of being involved in the continuity project.

The analysis process followed Braun and Clark’s^22^ six phases, and the first phase of the analysis was to become familiar with the data material, which began during the transcription of the interviews (BL). Thereafter, the material was read several times by researchers (BL and AK). During this first step, the second phase was initiated, and meaningful data extract was highlighted through the entire data set. Out of these data extracts, the initial codes were manually generated. All codes were then organized based on similarities into groups. A pattern arose, and during the third phase, preliminary themes and subthemes were generated. To visualize potential relationships between the themes, a thematic map was created. The initial coding, naming the themes and development of the thematic map were performed by the two authors conducting the interviews (BL and AK). During the fourth phase, the preliminary themes and subthemes were discussed and refined by all authors several times. Phase five, when the final titles for the themes and subthemes were defined, occurred simultaneously with phase four.

Close collaboration with the study participants was part of the research action design. This required that the researchers paid close attention based on their pre-understanding during the interviews and analysis process. All the researchers are midwives with academic degrees. The researchers who performed the interviews were not practically involved in the project or with the midwives, which allowed for an authentic dialogue. The only researcher with direct connection to the midwives (IH) took part in the analysis process after the thematic map was created where the individual midwives could not be identified. The study was approved by the regional ethics committee (Dnr. 2017/120-3).

## RESULTS

The analysis generated an overarching theme, ‘Developing a continuity model of midwifery care – demanding and rewarding with new insights’ and revealed a dualistic understanding described by the midwives who unwillingly had to leave a well-functioning work environment in the labor ward and, at the same time, received a unique opportunity to be part of a creative new midwifery model of care. The overarching theme consists of three themes and several subthemes. The three themes were: 1) A challenging but evolving start, 2) Varying views within the midwifery group, and 3) Visions for the future (Table 2).

**Table 2:** Overarching theme with themes and subthemes

*Developing a continuity model of midwifery care – demanding and rewarding with new insights*		
**A challenging but evolving start**	**Varying views within the midwifery group**	**Visions for the future**
Being in the shadow of a closure	Rural constraints and challenges	Extended availability
Creativity and optimism	Accessibility vs continuity	In-depth support when needed
Tailoring a new model	The midwife–woman relationship	Living the dream of reopening the labor ward

### A challenging but evolving start

This theme reflects the emotional impact from the closedown of the labor ward and how this, in turn, affected all the midwives in the project. It was considered a harsh blow to the families in the area since care during pregnancy, birth and postpartum was impaired. There was also grief over the loss of a well-functioning maternity service and the colleagues involved, which was a loss that had not fully been processed. The theme also considered organizational challenges and group processes based on the following three subthemes.

#### Being in the shadow of a closure

This subtheme mirrors the midwives’ conflicting feelings of involuntarily having to end a period in their working lives as midwives in intrapartum care. The closing of the labor ward was discussed with sorrow and anger. At the same time, there was a relief to be able to continue working in maternity care and engage in the full scope of the midwifery profession. Antenatal care was a new part of midwifery care for most of the midwives in the project, which was considered to be both challenging and difficult to learn. A positive aspect of being part of the project was that their place of work remained in their hometown. One of the midwives explained her feelings:

*‘On many levels, the closure implied great sorrow: losing our fine maternity ward and separation from the staff group. But I entered this [the project] with optimism.’* (Group interview 1)

#### Creativity and optimism

This subtheme reflects the feelings of pleasure expressed by the midwives to be part of the project and the start of something new. The offer to participate in the project was received with gratitude and joy. Being able to continue to work with parents-to-be and be part of a new model of midwifery care was satisfying. It was considered an easy choice and most fulfilling to be a midwife ‘all the way’ by following women through pregnancy and childbirth. The first months were characterized by enthusiasm and hard work, organizing everything from locations and equipment to training in new activities.

*‘Initially, it was blossoming! Creative, anything was possible. That's how it felt.’* (Midwife 1)

#### Tailoring a new model

The first round of interviews took place seven months after the start of the project, and all the midwives agreed about some important experiences. Decision making about almost everything required new independency, responsible thinking and creativity. The situation was described as both challenging and meaningful since, despite a hard workload, the midwives felt involved and fully participated in the building of the new continuity model. One midwife concluded:

*‘It was hard work and a lot of fun.’* (Group interview 2)

Alongside the positive expectations, doubts about the leadership arose. The management of the project was unclear, and the midwives lacked a leader for the project on-site. They had wanted more support from the management since the new routines and practical aspects to arrange sometimes became overwhelming. This was mirrored in the following quote:

*‘After a while, you felt a little drained of energy because there was so much [to manage], from the tiniest urine test strip to making things work organizationally and everything in between.’* (Group interview 1)

During the third and last interview session, when reflecting on the project, one midwife voiced that the greatness of such a work model is the freedom to make one’s own decisions and changes. However, a manager who was present on a daily basis facilitated the work and was able to observe the changes. Another opinion was, in retrospect, that external supervision could have made teambuilding smoother, as there was a new constellation within the work group.

*‘Then I believe that maybe you should also have someone, a behavioral scientist on the side, who could support this grieving process that we went through and the group process, as a matter of fact.’* (Midwife 2)

### Varying views within the midwifery group

Enthusiasm was the primary feeling regarding the working model experienced by the midwives. However, after working for some time in the project, which included long journeys to the labor wards, doubts about the benefits and sustainability of travel were raised. The theme consisted of the following three subthemes.

#### Rural constraints and challenges

When the on-call service for birth had been going on for some months, it became clear that the travel distance to the labor wards was a serious problem. It was discussed whether this model of continuity of midwifery care was the best way to support the women, as they had to travel 120 km one-way to the labor ward. The advantages with the continuity of midwifery model of care were, in general, well accepted and agreed upon among the midwives in the project. However, the limited midwife staffing affected the on-call service and the opportunities for the women to have a known midwife during labor. Aspects of security for the midwives were also raised when driving on slippery and dark roads in wintertime. These issues were viewed as vital during the interviews. Some of the midwives expressed that travelling back and forth to the maternity wards was a waste of time.

*‘It is too tiring and anxiety-provoking to be woken up in the middle of the night and drive 120 km in a car on icy roads. That feeling is not good, to be inefficient during working hours both heading to work and returning back home.’* (Midwife 3)

One aspect of the constraints in this rural area was the general negative opinion about the project from the local population when it started. The project was perceived as a bad substitute for the closure of the labor ward. In addition, the midwives felt they had to defend the project, which made them feel disloyal to the people in the area. The attitude towards the project shifted as time went by, and the opinion became more related to the women’s positive experiences of the continuity model. One of the midwives explained:

*‘But the resistance among people in the town was so great and that it, somehow, is such a strong force, and sometimes it is almost an evil force. This is what happens to people in a village when it becomes like this. [I never thought] that it would be so challenging and hit so hard.’* (Midwife 4)

#### Accessibility versus continuity

The opinions in the group of midwives varied, and discussions were on-going regarding whether a continuity model worked best when two midwives shared a caseload compared to a group of four to five midwives. However, they all agreed that the continuity model was a fruitful way to work. The midwives stressed that it meant freedom to plan the day and be familiar with the women they were about to meet. They also emphasized that a local birthing facility would have made a difference. Halfway through the project, the working conditions for the midwives shifted, which meant that the on-call service was reduced and, thereby, the possibility for continuity during birth. This was received with relief by some midwives and disappointment by others. Working the full scope of midwifery care that followed with the continuity model in the project was considered positive but did not compensate for the inconvenience of commuting. One midwife voiced:

*‘The basis for continuity, to get the whole picture, I really like it [….]. When you see all the parts and make the puzzle complete, it's so great. The distance to the hospitals is a great obstacle, having to travel, sitting in the car almost half the [working] time. Ooh, what a waste!’* (Midwife 1)

The diverse experiences among the midwives were also related to views about when women needed the project midwife the most. The importance of having a known midwife when giving birth was questioned since the interviewed midwives acknowledged that qualified midwifery care was already available at the labor wards. The limited on-call hours sometimes resulted in the birthing woman and her partner already being admitted to the labor ward. When this occurred, and the couples had already established a relationship with the ward midwife, some of the project midwives felt that their own presence became unnecessary. Some midwives voiced that their presence was more important in the rural town, for pregnant women and women that had recently given birth.

*‘… I was away [from the clinic] the whole day and there was no evening reception, while the patient I was with has a hospital full of people.’* (Midwife 5)

#### The midwife–woman relationship

The midwives expressed that the trust and relationship established during the antenatal visits was invaluable when the woman gave birth. Being familiar with the woman and her partner and knowing her family and history made all the difference during birth. The relationship with the woman made the midwife more sensitive towards the support needed. The core of continuity was to obtain an overall picture of the woman, her partner and their needs. In this aspect, the conclusion was that the known midwife was not that easily replaceable.

*‘And some things you do not even have to mention because I already know; they do not have to say it because we have talked about it, we have been on it. And all the work that can be done antenatally, with breathing exercises and relaxation.’* (Midwife 2)

### Visions for the future

During the process of the project, the midwives’ views of the future were developed. These ideas were expressed in the last interview sessions. The theme consisted of the following three subthemes.

#### Extended availability

When the project was about to end, the midwives discussed how the services for pregnant women and new parents in the rural area should continue in the future. They all agreed that increased accessibility should be offered and that midwives would be available for an extended reception during the daytime and evenings. The contact telephone was also mentioned as important, as women could have direct access to the midwives. The midwives were convinced that they should go on providing antenatal care, parental education and postnatal check-ups for both mother and baby, in addition to being widely accessible for women in the area. To facilitate the extended availability, the midwives pointed out that sufficient staffing was needed.

Moreover, the possibility to do home visits both before and after birth was expressed as being important. The midwives had all good experiences of seeing women early in labor for support during the latent phase, whether the water had already broken or the assessment of the onset of labor was underway. Equally important was to see the women after birth and provide practical support with breastfeeding and infant care. In addition, giving advice about physical and emotional changes after childbirth was perceived as easier when there was an ongoing relationship with the woman and her family. One quote to illustrate this was:

*‘I really believe we should have the opportunity to take in women and help things get under control if something is uncertain. This phone [used in the project] is worth its weight in gold, especially when antenatal care is closed in the evening and the wintertime. Should I go or not go and save someone from travelling unnecessarily and rather wait until tomorrow?’* (Midwife 5)

#### In-depth support when needed

Another matter that was voiced in the interviews was that there must be the possibility for a more individualized care for certain women, which also was the intention of the project from the start. The midwives perceived that some women needed to be offered more support, not only in terms of more antenatal visits but also support during labor and birth. One example that was mentioned was the young and single primipara afraid of giving birth who asked her midwife to assist her during labor.

#### Living the dream of reopening the labor ward

The midwives also argued for the need of a small birthing suite in the existing hospital. This would maintain the idea of the project and the continuity of care model. In addition, such an initiative would be welcome among the population and suitable for healthy multipara women with normal pregnancies and their families living in a rural area with a long distance to the nearest hospital. One midwife argued:

*‘… that you could have the opportunity to admit women who are in need after [the birth], in a postnatal place for observation for some time. It would be optimal to open a midwifery-led center and take these green-green [healthy] mothers here locally on a caseload model, so we could use this concept.’* (Midwife 5)

## DISCUSSION

The main findings of this study illuminate the demanding and rewarding process when introducing, implementing and evaluating a midwifery continuity model of care in a rural area after the closure of a labor ward.

The described three themes identified the midwives concerns when reflecting on the project. Initially, inclusion in the project was perceived challenging when experiencing contradictory feelings and had a pronounced impact on the midwives. On the one hand, grief and disappointment caused by the closure of the labor ward, without time for the grieving process, profoundly affected the midwives. At the time of closure, some of the staff at the labor ward lost their jobs. It is well known that work is a central and important part of people’s lives and existence, and losing a job may affect people’s mental health, their social relations and the economy^23^. On the other hand, being chosen to have the opportunity to create something completely new made the midwives happy and encouraged their creativity. There is a great body of research indicating that midwives highly value working within midwifery continuity models of care^2,24^.

Although the analysis showed that the midwives were positive about the creative process involved when tailoring a new model, they emphasized the lack of clear leadership when the model was about to start. There is always a balance between fostering autonomy and providing support. This requires strong leadership, as suggested by Styles et al.^25^ who concluded that the process of upscaling and implementing continuity models is facilitated through supportive midwifery, obstetric leadership, inter-disciplinary collaboration and rostering flexibility. Autonomy and the flexibility that comes with continuity models have been highly appreciated as evidenced by the literature^15,26^. It has also been shown that autonomy in work and passion for the profession could be protective factors against burnout^27-29^. A recent systematic review and meta-analysis of 8959 women confirmed that continuity models with on-call schedules were not associated with midwives’ burnout levels^7^.

Later in the process, it became obvious that the midwives became more critical towards the project. The criticism was mainly about the idea of continuity versus accessibility. These opinions were based primarily on the obstacles with the long travel distance and the limited time with a woman in labor due to the on-call hours that were not covered all day. This is in line with Redshaw et al.^30^ who describe how travelling long distances takes a significant toll on midwives’ time as well as having a profound impact on the caseload size. On the other hand, the midwives in the project emphasized that continuity was a good idea in theory, and that the midwife–woman relationship could ease the caring process, if there were a birthing facility nearby. More than two decades ago it was defined that a relationship between the midwife and the woman is therapeutic and ‘is at the core of human caring’^31^. However, in the discussions, some of the midwives disregarded the importance of this relationship with the view that competent midwifery care was available in the labor wards. This was mostly notable when the woman had gone into labor during the night and was already admitted to hospital when the on-call shift started, resulting in the midwives feeling less needed. This finding contradicts the results from Styles et al.^25^ where the interviewed midwives concluded that ‘even if the woman had only met the midwife attending her birth once, it would be better than having an unknown caregiver present for the birth’. It seems like some of the midwives did not fully encompass the idea of the relationship. The woman– midwife relationship is one of the cornerstones in continuity models, as shown by Perriman et al.^6^ in a meta-synthesis of 13 scientific articles about what women value in continuity models, e.g. personalized care, trust and empowerment. The relationship with the woman has also been acknowledged by midwives in several studies about continuity models^9,32,33^.

When the project in its present form was about to end, the midwives wanted to continue to be available on-site for extended hours. Some of the midwives also wanted to offer specific support to women they had identified as in need. A woman with fear of birth was brought up as an example of whom they believed would be better off with a known midwife during labor and birth. Previous studies have shown benefits with a known midwife, especially for women with fear of birth^34,35^. These studies show that it is achievable within the context of antenatal care or hospital-based clinics providing counselling to women with fear of birth^36^ to also offer women birth assistance from a midwife the women have previously met. This will eventually not only reduce women’s childbirth fear but erase the fear entirely^37^.

In the final interview, the grief of the closed labor ward had diminished to some degree. Nevertheless, the midwives lived the dream of re-opening the labor ward. Offering such alternatives for healthy multipara women was suggested as a means to increase safety and security and offer continuity. A problem in Sweden is the lack of alternative birth settings, despite evidence of their safety for selected women^38^. In another study from a rural area, a similar scenario was presented but with other solutions. In addition, birth services provided by midwives in collaboration with general practitioners with obstetric skills have been fruitful in rural Australia after closure of a rural birth service^16^. One of the reasons to re-open birth assistance was a belief that midwife-assisted births are safer than free births for healthy women in a rural area with a long distance to hospital.

The process throughout the project period could be understood in the light of Fisher’s Personal Transition Curve^39^. The process of change is always individual, and any change could create conflicts between existing values and beliefs and anticipations. It is important to understand people’s perceptions of the past, present and future and their coping abilities to support transitions. Fisher^39^ described certain stages in the change process, from anxiety to happiness, sometimes fear, threat and guilt when the individual is questioning herself, and further onward to gradual acceptance and moving forward. This is often the case when the transition process is straightforward. If not, the transition could result in disillusion or hostility. Some of these stages are clearly visible in the three interview sessions.

### Strengths and limitations

The strength of this study is the longitudinal approach, where the process of developing, launching and assessing a continuity of midwifery care project was based on the midwives’ perspective.

The participatory action research approach made it possible not only to change the model according to the midwives’ experiences, but also to develop a future model that could be sustainable within rural conditions. One limitation is that this approach, which led to a close collaboration between midwives and researchers, could have hampered the midwives from expressing their views during the interviews. However, according to the final data material and the midwives’ statements, this does not seem to be the case.

During the research process, several aspects have been considered to achieve trustworthiness^40^. The limited number of midwives in the project was compensated by the longitudinal design with repeated interviews with the participants during the project process, which ensured credibility. The interviews were also conducted in different ways, such as group, individual and telephone interviews, which strengthen credibility. The researchers’ experiences in midwifery and the collaborative sessions during the analysis phase further reinforced the credibility of the study. To enable other researchers to evaluate the relevance of the study, transferability was ensured through the detailed description of the study context, data collection and analysis of data. Dependability was reached by a rich description of the research process of the study, which allows the study to be repeated. Triangulation through the use of different interview methods, the researchers’ collaborative work and continuous discussions, and awareness of their preunderstanding ensured the confirmability of the study. In addition, the use of Braun and Clark’s^22^ checklist of criteria for good thematic analysis further reinforced confirmability.

## CONCLUSIONS

Some important lessons have been learned from the midwives’ experiences in the present study. The first lesson learned was that it is not convenient to introduce a new model of care shortly after closing a labor ward, when the midwives are grieving and in need of support themselves. The risk could be that the continuity model is viewed as a replacement of the closed labor ward and not as a way to provide high-quality evidence-based care.

The second lesson learned is that midwives who are attracted to work in continuity models need to understand and incorporate the prerequisites of such models and the midwifery group needs to strive for the same goal. Examples of important issues are the willingness to develop close relationships with the women and to be independently responsible to plan and organize their care.

The third lesson learned from the interviews with midwives is that continuity models, when long commuting to the hospital is required, need enough midwives to encourage safety and security for women at all times. The women’s improved care with continuity must be balanced against the midwives’ working conditions in order to be safe, sustainable and satisfying.
